# Role of fetal head‐circumference‐to‐maternal‐height ratio in predicting Cesarean section for labor dystocia: prospective multicenter study

**DOI:** 10.1002/uog.24981

**Published:** 2023-01-03

**Authors:** A. Dall'Asta, R. Ramirez Zegarra, E. Corno, I. Mappa, J. L. A. Lu, E. Di Pasquo, G. Morganelli, M. Abou‐Dakn, C. Germano, R. Attini, B. Masturzo, G. Rizzo, T. Ghi

**Affiliations:** ^1^ Department of Medicine and Surgery, Obstetrics and Gynecology Unit University of Parma Parma Italy; ^2^ Department of Obstetrics and Gynecology St Joseph Krankenhaus Berlin Germany; ^3^ Department of Obstetrics and Gynecology, Fondazione Policlinico Tor Vergata University of Rome Tor Vergata Rome Italy; ^4^ Department of Obstetrics and Gynecology, Sant'Anna Hospital University of Turin Turin Italy

**Keywords:** Cesarean section, head circumference, labor dystocia, maternal height, vaginal delivery

## Abstract

**Objective:**

To evaluate the relationship between the fetal head‐circumference‐to‐maternal‐height (HC/MH) ratio measured shortly before delivery and the occurrence of Cesarean section (CS) for labor dystocia.

**Methods:**

This was a multicenter prospective cohort study involving four tertiary maternity hospitals. An unselected cohort of women with a singleton fetus in cephalic presentation, at a gestational age beyond 36 + 0 weeks and without any contraindication for vaginal delivery, was enrolled between September 2020 and November 2021. The MH and fetal HC were measured on admission of the patient to the labor ward. The primary outcome of the study was the performance of the HC/MH ratio in the prediction of CS for labor dystocia. Women who underwent CS for any indication other than failed labor progression, including fetal distress, were excluded from the final analysis.

**Results:**

A total of 783 women were included in the study. Vaginal delivery occurred in 744 (95.0%) women and CS for labor dystocia in 39 (5.0%). CS for labor dystocia was associated with shorter MH (mean ± SD, 160.4 ± 6.6 *vs* 164.5 ± 6.3 cm; *P* < 0.001), larger fetal HC (339.6 ± 9.5 *vs* 330.7 ± 13.0 mm; *P* < 0.001) and a higher HC/MH ratio (2.12 ± 0.11 *vs* 2.01 ± 0.10; *P* < 0.001) compared with vaginal delivery. Multivariate logistic regression analysis showed that the HC/MH ratio was associated independently with CS for labor dystocia (adjusted odds ratio, 2.65 (95% CI, 1.85–3.79); *P* < 0.001). The HC/MH ratio had an area under the receiver‐operating‐characteristics curve of 0.77 and an optimal cut‐off value for discriminating between vaginal delivery and CS for labor dystocia of 2.09, which was associated with a sensitivity of 0.62 (95% CI, 0.45–0.77), specificity of 0.79 (95% CI, 0.76–0.82), positive predictive value of 0.13 (95% CI, 0.09–0.19) and negative predictive value of 0.98 (95% CI, 0.96–0.99).

**Conclusions:**

In a large cohort of unselected pregnancies, the HC/MH ratio performed better than did fetal HC and MH alone in identifying those cases that will undergo CS for labor dystocia, albeit with moderate predictive value. The HC/MH ratio could assist in the evaluation of women at risk for CS for labor dystocia. © 2022 The Authors. *Ultrasound in Obstetrics & Gynecology* published by John Wiley & Sons Ltd on behalf of International Society of Ultrasound in Obstetrics and Gynecology.

## Abstract 2

This article's abstract has been translated into Spanish and Chinese. Follow the links from the abstract to view the translations.

## RESUMEN

**Función de la relación entre el perímetro cefálico del feto y la altura de la madre en la predicción de la cesárea por distocia del parto: estudio multicéntrico prospectivo**

**Objetivo:**

Evaluar la relación entre el perímetro cefálico del feto y la altura materna (PC/AM) medidos poco antes del parto y la incidencia de cesárea (CS) por distocia del parto.

**Métodos:**

El tipo de estudio fue de cohortes prospectivo multicéntrico en el que participaron cuatro hospitales materno‐infantiles de tipo terciario. Entre septiembre de 2020 y noviembre de 2021se alistó una cohorte no seleccionada de mujeres con feto único en presentación cefálica, a una edad gestacional superior a las 36+0 semanas y sin ninguna contraindicación para el parto vaginal. La AM y el PC del feto se midieron en el momento del ingreso de la paciente a la sala de partos. El resultado primario del estudio fue la eficacia del cociente PC/AM en la predicción de la CS por distocia del parto. Se excluyeron del análisis final las mujeres a las que se practicó una cesárea por cualquier otra indicación que no fuera el fracaso del proceso del parto, incluido el sufrimiento fetal.

**Resultados:**

En total, se incluyeron 783 mujeres en el estudio. El parto vaginal se produjo en 744 (95,0%) mujeres y la cesárea por distocia del parto en 39 (5,0%). La cesárea por distocia del parto se asoció con una AM más baja (media ± DE, 160,4 ± 6,6 frente a 164,5 ± 6,3 cm; P < 0,001), mayor PC del feto (339,6 ± 9,5 frente a 330,7 ± 13,0 mm; P < 0,001) y un mayor cociente PC/AM (2,12 ± 0,11 frente a 2,01 ± 0,10; P < 0,001) en comparación con el parto vaginal. El análisis de regresión logística multivariante mostró que el cociente PC/AM se asoció de forma independiente con la CS por distocia del parto (razón de momios ajustada, 2,65 [IC 95%, 1,85–3,79]); P < 0,001). El cociente PC/AM tuvo un área bajo la curva de características operativas del receptor de 0,77 y un valor de corte óptimo para discriminar entre parto vaginal y CS por distocia del parto de 2,09, que se asoció a una sensibilidad de 0,62 (IC 95%, 0,45–0,77), una especificidad de 0,79 (IC 95%, 0,76–0,82), un valor predictivo positivo de 0,13 (IC 95%, 0,09–0,19) y un valor predictivo negativo de 0,98 (IC 95%, 0,96–0,99).

**Conclusiones:**

En una gran cohorte de embarazos no seleccionados, el cociente PC/AM funcionó mejor que el PC del feto y la AM por sí solos a la hora de identificar los casos que se someterán a una cesárea por distocia del parto, aunque con un valor predictivo moderado. El cociente PC/AM podría ayudar en la evaluación de las mujeres con riesgo de cesárea por distocia del parto.

## 摘要

胎儿头围与母亲身高比值在预测因难产而实施剖腹产中的作用:前瞻性多中心研究

**目的** 评价分娩前不久测量的胎儿头围与母亲身高(HC/MH)比值与因难产而进行剖腹产(CS)之间的关系。

**方法** 这是一项多中心的前瞻性队列研究，涉及四家三级妇产医院。在2020年9月至2021年11月期间，招募了一个未经选择的队列，队列中的产妇为单胎头位，胎龄超过36+0周，没有任何阴道分娩的禁忌症。在患者入院时，对MH和胎儿HC进行了测量。研究的主要结果是HC/MH比值在预测因难产而实施剖腹产中的表现。最终分析中不包括因产程进展失败以外的任何指征(包括胎儿窘迫)而接受CS的产妇。

**结果** 共有783名产妇被纳入研究。744名(95.0%)产妇经阴道分娩，39名(5.0%)产妇因难产进行了剖腹产。与阴道分娩相比，因难产进行的剖腹产与较小的MH(平均±SD，160.4±6.6 vs 164.5±6.3 cm；P<0.001)、较大的胎儿HC(339.6±9.5 vs 330.7±13.0 mm；P<0.001)和较高的HC/MH比值(2.12±0.11 vs 2.01±0.10；P<0.001)有关。多变量逻辑回归分析显示，HC/MH比值与因难产而进行的CS独立相关(调整后的优势比，2.65(95%CI，1.85‐3.79)；P<0.001)。HC/MH比值在接受者操作特征曲线下的面积为0.77，区分阴道分娩和因难产而进行的CS的最佳截止值为2.09，其敏感性为0.62(95%CI，0.45‐0.77)，特异性为0.79(95%CI，0.76‐0.82)，阳性预测值为0.13(95%CI，0.09‐0.19)和阴性预测值为0.98(95%CI，0.96‐0.99)。

**结论** 在一个未经选择的大队列中，HC/MH比值在识别那些因难产而接受CS的病例方面比单独的胎儿HC和MH表现更好，尽管预测价值不高。HC/MH比值可协助评估产妇因难产而接受CS的风险。

**CONTRIBUTION**

*What are the novel findings of this work?*

The newly described fetal head‐circumference‐to‐maternal‐height (HC/MH) ratio performs better than do fetal HC and MH alone in identifying cases that will undergo Cesarean section for labor dystocia, albeit with moderate predictive value.

*What are the clinical implications of this work?*

In an unselected population at low risk for labor dystocia enrolled within 72 h before delivery, the HC/MH ratio demonstrates a fair specificity and negative predictive value for vaginal delivery. Use of the HC/MH ratio could assist in the evaluation of women at risk for Cesarean section for labor dystocia.

## INTRODUCTION

Labor dystocia is estimated to account for half of all primary Cesarean sections (CS)^1^ and represents a risk factor for maternal and neonatal morbidity^2^, ^3^, ^4^. Labor dystocia may result from a relative or absolute mismatch between the size of the birth canal and that of the fetus^5^. The identification of women at risk for obstructed labor is among the main, and so far, unresolved, goals of antenatal evaluation.

Over the years, several studies have investigated the role of maternal and fetal anthropometric characteristics, such as maternal height (MH)^6^, ^7^, ^8^, ^9^ and fetal head circumference (HC)^10^, ^11^, ^12^, ^13^, ^14^ and size^15^, ^16^, ^17^, ^18^, in determining the risk of labor dystocia leading to CS. MH can be evaluated easily and has been highlighted as a possible surrogate for the size of the birth canal^19^, ^20^ and an indicator of the risk of CS secondary to labor dystocia^6^, ^7^, ^8^, ^9^. With respect to fetal anthropometrics, as the fetal head must negotiate the maternal pelvic inlet, a large fetal HC has been proposed as a risk factor for labor dystocia leading to CS^10^, ^11^, ^12^, ^13^, ^14^. Nonetheless, to the best of our knowledge, no studies have evaluated a combination of maternal and fetal anthropometrics in relation to the risk of CS secondary to labor dystocia. We propose the ratio between the fetal HC and the MH (HC/MH ratio) as a more reliable predictor of obstructed labor compared with the constituent parameters on their own. The aim of this study was to evaluate the relationship between the HC/MH ratio calculated shortly before delivery and the occurrence of CS for labor dystocia.

## METHODS

This was a multicenter prospective observational study conducted at four tertiary maternity hospitals, in Italy (University Hospitals of Parma, Rome Tor Vergata and Turin) and Germany (St Joseph Krankenhaus, Berlin), between September 2020 and November 2021. A non‐consecutive series of singleton pregnancies with a cephalic‐presenting fetus, gestational age (GA) ≥ 36 + 0 weeks and no contraindications for vaginal delivery were considered eligible for the study. Patients were approached on admission due to labor induction, premature rupture of the membranes or spontaneous labor. Informed consent for study participation was obtained upon enrolment. In all included cases, the fetal HC was measured on transabdominal ultrasound in the standard transventricular plane^21^, including the fetal scalp surrounding the fetal calvaria, within 72 h before delivery. Women scheduled for elective CS were excluded from the study.

A common protocol for labor management was shared across the participating units during the study period. Labor dystocia was defined based on the American College of Obstetricians and Gynecologists (ACOG)/Society for Maternal–Fetal Medicine (SMFM) recommendations for the safe prevention of primary CS^22^. In detail, a protracted active phase of labor was defined as ≥ 6 cm of dilatation with ruptured membranes and failure to progress despite 4 h of adequate uterine activity or at least 6 h of oxytocin administration with inadequate uterine activity. Arrest of dilatation in the first stage requiring CS was diagnosed following two more hours of oxytocin administration with no cervical change. A diagnosis of arrest of labor in the second stage was made in the event that the duration of the active phase was at least 2 h in parous women and 3 h in nulliparous women on epidural analgesia, or 1 h and 2 h in parous and nulliparous women without epidural analgesia, respectively.

According to the local protocol of the participating units, women diagnosed with labor dystocia underwent clinical examination by the senior obstetrician responsible for patient care. Obstetric intervention for labor dystocia was performed when the criteria for arrest of dilatation or arrest of labor in the second stage were fulfilled^22^. The obstetricians in charge of labor care were blinded to the measurement of fetal HC shortly before birth. Cases in which CS was performed for any reason other than labor dystocia, such as suspected intrapartum fetal distress, were excluded from data analysis.

Clinical data including maternal age, ethnicity, parity, height and body mass index (BMI), as well as GA at inclusion, mode of delivery and indications for CS, and neonatal outcomes, including birth weight, umbilical artery pH and 5‐min Apgar score, were extracted from patient case notes. The HC/MH ratio was computed as the ratio between the measurement of the HC, expressed in mm, and that of the MH, expressed in cm. All information was collected after birth using a dedicated form. Data were recorded and stored in a Microsoft Excel secured pseudonymized database (Microsoft Corp., Redmond, WA, USA), which was accessible only to members of the research team. The primary outcome of the study was the relationship between the HC/MH ratio within 72 h before birth and the occurrence of CS for labor dystocia.

Statistical analysis was performed using Statistical Package for Social Sciences (SPSS) version 22 (IBM Corp., Armonk, NY, USA). The Kolmogorov–Smirnov test was used to assess the normality of the distribution of the data. The chi‐square test was used to compare categorical variables and Student's *t*‐test or the Mann–Whitney *U*‐test was used as appropriate for continuous variables. Results are presented as *n* (%), mean ± SD or median (range). Logistic regression analysis was used to control for potential confounding variables. The performance of MH, HC and the HC/MH ratio in predicting CS for labor dystocia was determined using receiver‐operating‐characteristics (ROC)‐curve analysis. The DeLong method was used for the comparison of areas under the ROC curve (AUCs). *P* < 0.05 was considered statistically significant. The study was approved by the local ethics committee of each participating unit and was reported in accordance with strengthening the reporting of observational studies in epidemiology (STROBE) guidelines^23^.

## RESULTS

**Figure 1:**
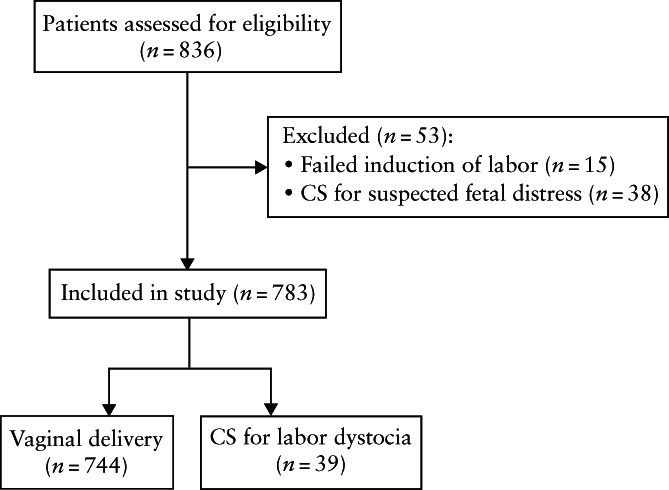
STROBE flowchart summarizing inclusion of women with singleton pregnancy beyond 36 + 0 weeks of gestation who were admitted to labor ward and had no contraindication for vaginal delivery. CS, Cesarean section.

Overall, 836 women were eligible over the study period. Of these, 15 were excluded due to failed induction of labor and 38 due to CS for suspected intrapartum fetal distress, leaving 783 cases for data analysis (Figure 1). Among the included cases, vaginal delivery occurred in 744 (95.0%) women and CS for labor dystocia in 39 (5.0%).

The demographic and perinatal characteristics of the study population overall are shown in Table 1 and are stratified according to the mode of delivery in Table 2. CS for labor dystocia was associated with higher BMI at term (mean ± SD, 29.4 ± 4.9 *vs* 27.7 ± 4.1 kg/m^2^; *P* = 0.01), higher frequency of nulliparity (79.5% *vs* 52.7%; *P* = 0.001) and epidural use (74.4% *vs* 43.4%; *P* < 0.001), higher GA at inclusion (40 + 1 ± 1 + 0 *vs* 39 + 4 ± 1 + 2 weeks; *P* = 0.007), larger fetal HC (339.6 ± 9.5 *vs* 330.7 ± 13.0 mm; *P* < 0.001), higher HC/MH ratio (2.12 ± 0.11 *vs* 2.01 ± 0.10; *P* < 0.001), higher birth weight (3504.7 ± 405.6 *vs* 3357.5 ± 449.4 g; *P* = 0.04) and shorter MH (160.4 ± 6.6 *vs* 164.5 ± 6.3 cm; *P* < 0.001), compared with vaginal delivery.

**Table 1:** Baseline characteristics and perinatal outcome of 783 included cases

Characteristic	Value
Maternal age (years)	31.7 ± 5.2
BMI at presentation (kg/m^2^)	23.3 ± 4.3
BMI at term (kg/m^2^)	27.8 ± 4.2
MH (cm)	164.3 ± 6.3
Nulliparous	423 (54.0)
GA at inclusion (weeks)	39 + 5 ± 1 + 2
Induction of labor	311 (40.0)
Epidural use	352 (45.0)
Fetal HC (mm)	331.1 ± 13.0
HC/MH ratio	2.02 ± 0.10
Birth weight (g)	3364.9 ± 448.3
5‐min Apgar score	10 (6–10)
Umbilical artery pH	7.24 ± 0.08

Data are given as mean ± SD, *n* (%) or median (range).

BMI, body mass index; GA, gestational age; HC, head circumference; MH, maternal height.

**Table 2:** Baseline characteristics and perinatal outcome of 783 included cases, according to mode of delivery

	Vaginal delivery	CS for labor dystocia	
Characteristic	(*n* = 744)	(*n* = 39)	*P*
Maternal age (years)	31.6 ± 5.3	32.3 ± 4.6	0.43
BMI at presentation (kg/m^2^)	23.3 ± 4.3	23.8 ± 4.6	0.45
BMI at term (kg/m^2^)	27.7 ± 4.1	29.4 ± 4.9	0.01
MH (cm)	164.5 ± 6.3	160.4 ± 6.6	< 0.001
Nulliparous	392 (52.7)	31 (79.5)	0.001
GA at inclusion (weeks)	39 + 4 ± 1 + 2	40 + 1 ± 1 + 0	0.007
Induction of labor	295 (39.7)	16 (41.0)	0.78
Epidural use	323 (43.4)	29 (74.4)	< 0.001
Fetal HC (mm)	330.7 ± 13.0	339.6 ± 9.5	< 0.001
HC/MH ratio	2.01 ± 0.10	2.12 ± 0.11	< 0.001
Birth weight (g)	3357.5 ± 449.4	3504.7 ± 405.6	0.04
5‐min Apgar score	10 (6–10)	10 (8–10)	0.76
Umbilical artery pH	7.24 ± 0.08	7.26 ± 0.09	0.18

Data are given as mean ± SD, *n* (%) or median (range).

BMI, body mass index; CS, Cesarean section; GA, gestational age; HC, head circumference; MH, maternal height.

**Figure 2:**
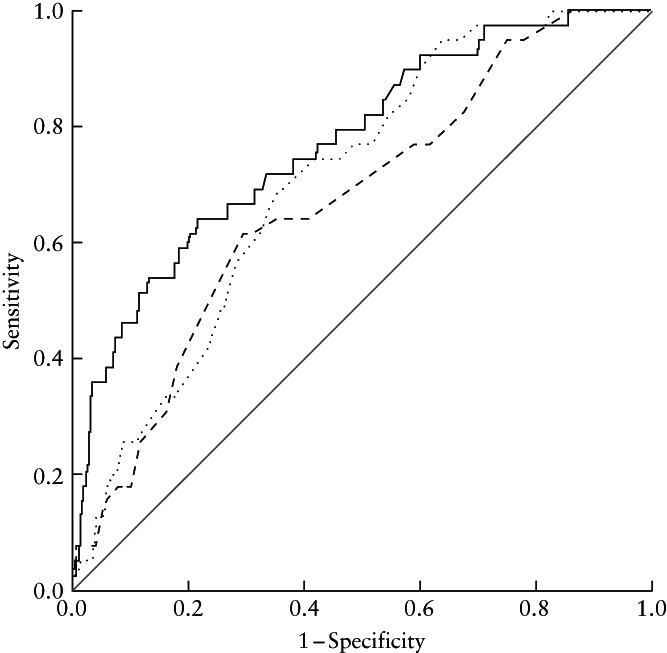
Receiver‐operating‐characteristics curves for maternal height (MH) (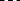), fetal head circumference (HC) (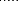) and HC/MH ratio (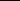) in prediction of Cesarean section for labor dystocia.

Logistic regression analysis showed that the HC/MH ratio (adjusted odds ratio (aOR), 2.65 (95% CI, 1.85–3.79); *P* < 0.001), MH (aOR, 0.89 (95% CI, 0.84–0.94); *P* < 0.001), fetal HC (aOR, 1.06 (95% CI, 1.03–1.10); *P* < 0.001), parity (aOR, 0.41 (95% CI, 0.17–0.98), *P* = 0.04) and epidural use (aOR, 2.43 (95% CI, 1.08–5.46); *P* = 0.03) were associated independently with CS for labor dystocia (Table 3). The AUCs of MH and fetal HC for predicting CS for labor dystocia were 0.67 (95% CI, 0.59–0.76; *P* < 0.001) and 0.71 (95% CI, 0.64–0.77; *P* < 0.001), respectively, while the HC/MH ratio had the highest AUC (0.77 (95% CI, 0.70–0.85); *P* < 0.001). This was higher than that of the MH and of the fetal HC alone (*P* < 0.001 and *P* = 0.04, respectively) (Figure 2). The optimal cut‐off value for discriminating between vaginal delivery and CS for labor dystocia was 2.09, and was associated with a sensitivity of 0.62 (95% CI, 0.45–0.77), specificity of 0.79 (95% CI, 0.76–0.82), positive predictive value (PPV) of 0.13 (95% CI, 0.09–0.19) and negative predictive value (NPV) of 0.98 (95% CI, 0.96–0.99).

**Table 3:** Logistic regression analysis for association of baseline characteristics with Cesarean section for labor dystocia

Characteristic	aOR (95% CI)	*P*
HC/MH ratio	2.65 (1.85–3.79)	< 0.001
MH	0.89 (0.84–0.94)	< 0.001
Fetal HC	1.06 (1.03–1.10)	< 0.001
Parity	0.41 (0.17–0.98)	0.04
Epidural use	2.43 (1.08–5.46)	0.03
BMI at term	1.07 (0.99–1.16)	0.11

aOR, adjusted odds ratio; BMI, body mass index; HC, head circumference; MH, maternal height.

## DISCUSSION

### Main findings

This study demonstrates that the HC/MH ratio is associated independently with CS for labor dystocia. The HC/MH ratio had the greatest accuracy in identifying cases that would undergo CS for labor dystocia, albeit with poor predictive value in our population of women at low risk for labor dystocia leading to CS.

### Interpretation of findings and comparison with previous studies

Several studies suggest an association between MH and mode of delivery, including CS for labor dystocia^6^, ^7^, ^8^, ^9^. This may be due to the fact that short women tend to have a proportionately narrow pelvis^19^, ^20^, however, environmental factors which are recognized determinants of MH may also impact the occurrence of adverse labor outcome^24^, ^25^. Another proposed explanation is that short women are more likely to be overweight or obese^26^, ^27^. Our data confirm the association between short MH and CS for labor dystocia. Previous studies evaluating antenatal pelvimetry on ultrasound^28^, ^29^, ^30^ or magnetic resonance imaging (MRI)^31^, ^32^ have demonstrated improved performance of direct pelvimetry compared with indirect assessment, as is the case with MH.

The influence of fetal size on labor outcome has also been studied extensively^15^, ^16^. A high estimated fetal weight (EFW) represents a risk factor for CS for labor dystocia^17^, ^18^, however prenatal ultrasound assessment of EFW has been shown to have limited accuracy in the prediction of fetal overgrowth^33^, ^34^, ^35^. Recent studies have suggested that the fetal HC represents a more reliable predictor of obstructed labor compared with EFW^10^, ^11^, ^12^, ^13^, ^14^, ^36^. Our results demonstrate that the HC/MH ratio performs better than MH and fetal HC alone in predicting CS for labor dystocia, thus supporting the theory that labor dystocia is not an isolated maternal‐ or fetal‐driven phenomenon, but may result from the interaction between maternal and fetal anthropometrics.

### Clinical implications and future perspectives

The present study suggests that predictive parameters or models aiming to identify those laboring women at risk for labor dystocia leading to CS should ideally include maternal and fetal anthropometric characteristics.

Our results demonstrate a fair specificity and NPV of the HC/MH ratio, meaning that women with a HC/MH ratio below the index threshold have a good chance of delivering vaginally. Nonetheless, our data indicate that the HC/MH ratio has a poor sensitivity and PPV for CS for labor dystocia. On one hand, this can be explained by the fact that the size of the fetus and the pelvis do not represent the only determinants of obstructed labor, as malposition, cephalic malpresentation and asynclitism are among the factors which may cause a relative mismatch between size of the fetus and that of the birth canal^37^, ^38^, ^39^. On the other hand, our results need to be interpreted in the context of the study population, who were at low risk for labor dystocia. Therefore, the actual performance of the HC/MH ratio in predicting CS for labor dystocia should be investigated in future prospective studies in women diagnosed with labor dystocia. In such cases, the prolongation of labor has been shown to be detrimental in terms of maternal and neonatal outcome^2^, ^3^, ^4^. Should our hypothesis that the HC/MH ratio could assist in deciding the mode of delivery in the context of labor dystocia be confirmed, then the HC/MH ratio may represent a useful and readily available tool with the potential to improve labor management and outcome.

Moreover, the HC/MH ratio may also support obstetricians evaluating women at risk for fetal macrosomia close to term. A randomized controlled trial demonstrated that labor induction before 39 gestational weeks improved labor outcome in women at risk for fetal macrosomia based on an EFW above the 95^th^ percentile for the given GA^40^. However, the antenatal prediction of fetal macrosomia on two‐dimensional^33^, ^34^ or three‐dimensional^41^, ^42^, ^43^ ultrasound has shown poor accuracy, while fetal weight estimation by MRI^44^ is unlikely to be readily available in an emergency setting. In this context, the HC/MH ratio may be superior to the ultrasound estimation of fetal weight in identifying those women who may benefit from early labor induction. This hypothesis also warrants investigation in prospective studies.

### Strengths and limitations

The strengths of this study include its evaluation of a novel parameter and the prospective enrolment of a large cohort of cases. The HC/MH ratio demonstrated fair specificity and NPV for identifying cases with a good chance of vaginal delivery, which may have potential utility in the context of labor dystocia and the antenatal counseling of patients with a large‐for‐gestational‐age fetus. Another advantage of the parameter is that it can be quantified easily and quickly, and hence be readily available, even in emergency settings.

Conversely, the non‐consecutive enrolment of patients could be seen as a limitation of the study findings. Furthermore, our results may not be generalizable to all centers, given that the study was conducted across four academic centers sharing a common protocol for labor management. The inclusion of the fetal scalp in the measurement of fetal HC could be also seen as a limitation, as it is not the standard recommendation^21^. However, we decided to include the fetal scalp in our measurement because we were not interested in brain growth, but rather in the entire size of the fetal head. Finally, fetal malposition, head malpresentation and asynclitism as determinants of obstructed labor in the presence of favorable maternal and fetal anthropometrics^45^, ^46^, ^47^, ^48^, ^49^, ^50^, ^51^ were not evaluated in this study.

### Conclusions

In a large cohort of women at low risk of labor dystocia enrolled within 72 h before delivery, the HC/MH ratio performed better than did fetal HC and MH alone in identifying those cases that will undergo CS for labor dystocia, albeit with poor predictive value. Further studies are needed to evaluate the performance of the HC/MH ratio in high‐risk settings, such as in the event of labor dystocia or antenatal suspicion of fetal macrosomia.

## Data Availability

The data that support the findings of this study are available on request from the corresponding author. The data are not publicly available due to privacy or ethical restrictions.
